# Dr. Kinglake, on Gout, in Reply

**Published:** 1805-06-01

**Authors:** Robert Kinglake

**Affiliations:** Taunton


### *To the Editors of the Medical and Physical Journal.*

Gentlemen,

The Remarks of your Correspondent signed S. on Mr. O'Neal's case and myself, in your last Journal, are from a Dr. Scott, of Newbury in Berkshire. The Doctor has enabled me to furnish the public with his signature, by transmitting me a counterpart of the letter which he had *previously* sent for insertion in your Journal.

The Doctor was induced to put me in possession of that performance by a motive of (what he terms) *candour*. That sentiment the Doctor might have felt, indeed, in his own *peculiar* way, but, in my estimation, it savours much more of malevolence than of that gentle virtue. The public will also probably look through the Doctor's letter in vain for his his boasted *candour*. No remark appears in this letter that was not anticipated in my observations, subjoined to Mr. O'Neal's Case.*

Dr. Scott speaks of repelled gout, insists on its destructive influence in Mr. O'Neal's case, and protests against apoplectic affection having existed. All these are gratuitous assertions. He then politely tells the public, he does not understand my theory of gout, and that to many sentences in my publication he can attach no meaning. This must be attributed either to the Doctor's dulness or to my obscurity. Here we are fairly at issue; and the Doctor will, perhaps, have to seek his defence by presenting the public with a production much less common-placed and hypothetical than the observations contained in his letter.

The refrigerant treatment of gout continues to succeed in the most promising manner, and that even under circumstances of the most tremulous atony. It is under the sure guidance of the natural sensation of heat and cold, and expressly concedes every indulgence with respect to temperature to the changeful feelings of patients. Where a sense of coldness is felt at the stomach during the treatment, it is probably induced by the sympathetic influence of a chilled skin from the impression of local cold. It rarely indeed occurs; but when it happens, there is the same occasion for exhibiting a stomachic cordial, whether domestic or medicinal, as there is for inviting augmented heat to the surface by either additional cloathing or fire. This is the practice authorised by my theory of gout, and is particularly enjoined by my dissertation on that disease. Were a patient suffering under local inflammation from fracture, contusion, incision, or any other injury from external violence, to be chilled by an excessive application of cold water to the affected part; its effect (as in gout) may be announced, either on the stomach by sickness and a sense of coldness in that organ, or on the brain in the form of stupor. These are symptoms of diminished vital energy, and will yield to moderate excitement, on the same evident principle that benumbed fingers from extreme cold become genially warmed by increased heat. The parallel likewise holds good in the mode in which relief is obtained by additional

---

* See Medical and Physical Journal, No. 73, page 231.

excitement. If attempted by gentle means, it might be effected; but if solicited by abrupt and immoderate stimulation, the sinking energies of life will be more likely to be mortally exhausted, than salutarily recruited by such undue violence.

Dr. Scott puts questions relative to the previous and subsequent general feelings of gouty persons in a gouty attack, and assumes as a fact, that the stomach is almost invariably affected before the local excitement happens on the joints. That this organ is occasionally disturbed by dyspeptic symptoms, before the appearance of inflammatory swellings on the joints, is too notorious to be questioned; but my theory of gout accounts for that circumstance, by considering indigestion as a leading remote cause of the disease. If a morbid susceptibility for inflammatory affection should exist in the ligamentous and tendinous structure, the systematic derangement induced by the occurrence of indigestion, will dispose that fabric more particularly to diseased excitement; hence the cause and effect which occasionally link together indigestion and gout. But the Doctor says, indigestion is relieved by the local inflammation. Here he deserts the ground of fact, and launches into the regions of fiction, in a manner (to retaliate on the Doctor) not at all comprehensible to my understanding.

The gouty sufferer can best decide, whether either the occurrence or duration of the inflammatory excitement of the joints *charms* away indigesiton, or whether it does not rather generate, exasperate, and prolong that debilitating evil. It is, indeed, not yet to enquire if digestion be compatible with exquisite pain from whatever cause it may arise. If the pulse be agitated by severe torture, however it may be named, the stomach will sympathise too acutely in the suffering to be capable of salutary digestion, nor will that alimentary process return but with the cessation of the pain that disordered it.

Your readers' time shall not be farther trespassed on by repeating remarks already extant, on a subject fairly before the public for experimental trial. The treatment proceeds prosperously, and justifies my theory, which, whether intelligible to Dr. Scott or not, importantly avails in diminishing the affliction, and improving the health of mankind. Its practical application is equally safe and precise, if directed by the unerring guide of sensual temperature.

Dr. Scott will, of course, exempt me from any charge of breach breach of trust in publishing his name; not indeed expressly confided to me, but given under a pretence of *candour,* not in my power either to conciliate or acknowledge; nor does any admissible reason appear why the Doctor should disclose to me his whole signature, and limit the public to a single initial of it. The Doctor should not make known under the letter S. what he would not unreservedly support by his entire name.

I am, &c.

*Taunton, April* 14, 1805. ROBERT KINGLAKE.

